# Development of Validated and Stability-Indicating LC-DAD and LC-MS/MS Methods for Determination of Avanafil in Pharmaceutical Preparations and Identification of a Novel Degradation Product by LCMS-IT-TOF

**DOI:** 10.3390/molecules23071771

**Published:** 2018-07-19

**Authors:** Nafiz Öncü CAN

**Affiliations:** 1 Department of Analytical Chemistry, Faculty of Pharmacy, Anadolu University, Eskişehir 26470, Turkey; nafizoc@anadolu.edu.tr; Tel.: +90-532-556-6824; 2 Doping and Narcotic Compounds Analysis Laboratory, Faculty of Pharmacy, Anadolu University, Eskişehir 26470, Turkey

**Keywords:** avanafil, stability-indicating assay method, novel degradation product, monolithic silica column, high-resolution mass spectrometry, LCMS-IT-TOF, LC-MS/MS, LC-DAD

## Abstract

Avanafil (AVA), one of the most effective drugs prescribed for erectile dysfunction, is a pyrimidine-derivative PDE5 inhibitor. In the current work, new LC methods were developed and validated for quantitative determination of avanafil and qualitative determination of its degradation products. The quantitative determination of avanafil was carried out using liquid chromatography with photodiode array detection (LC-DAD) and liquid chromatography-tandem mass spectrometry LC-MS/MS methods, and fully validated according to the ICH Q2 (R1) guideline, while qualitative determination was performed using a liquid chromatography mass spectrometry-ion trap-time of flight (LCMS-IT-TOF) instrument. The separation of avanafil and its degradation products was carried out using the same reversed-phase chromatographic conditions, in which a second-generation C_18_-bonded monolithic silica column (Chromolith^®^ High Resolution RP-18e, 100 × 4.6 mm, Merck KGaA) was used as stationary phase. Briefly, the methods enable quantitation of avanafil with high accuracy (recovery > 95%) and precision (RSD% < 2.0), within the ranges of 0.5–20 μg/mL for LC-DAD and 150–6000 ng/mL for LC-MS/MS. In the forced degradation studies, over and above currently existing data, a new oxidation-based degradation product, whose predicted *m/z* is 367.1168, was identified and its structure was confirmed by high-resolution mass spectrometric analysis. As the main advantage, either an LC-DAD or LC-MS/MS instrument can be chosen for interference-free quantitation of AVA, according to the facilities in quality-control laboratories.

## 1. Introduction

**Figure 1:**
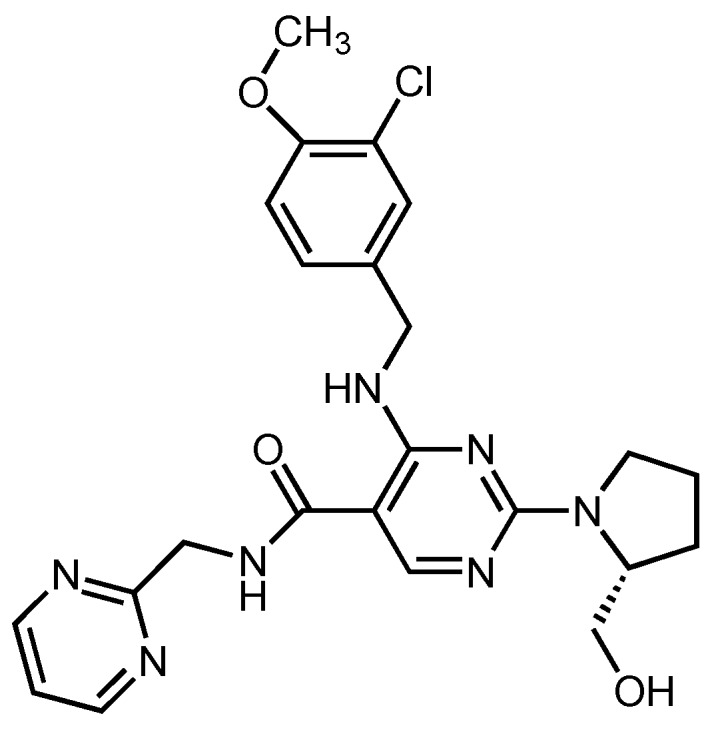
The chemical structure of AVA.

Erectile dysfunction (ED), one of the common sexual disturbances in the adult male, is described as a permanent or repetitive inability to attain and/or maintain penile erection for satisfactory sexual performance [1]. It has been estimated that 5–20% of men are affected by moderate-to-severe ED at some time during their sexual life [2]. Several phosphodiesterase-5 (PDE5) inhibitors are in use for treatment of ED such as sildenafil, vardenafil, tadalafil, avanafil (AVA) and udenafil. Among these, AVA rapidly acts and shows low visual side effects compared to the other PDE5 inhibitors, and so it is used commonly for erectile dysfunction treatment (Figure 1) [3].

Approval of AVA by the US Food and Drug Administration was realized in 2012, and it was followed by European Medicines Agency in 2013. Studies to date have demonstrated that AVA has a time to maximum plasma concentration of 35–45 min, an elimination half-life less than 1.5 h, and 67–72% of patients successfully complete intercourse within 15 min of administration; specific inhibition of PDE5 activity at a 50% inhibitory concentration of AVA was 4.3 nM with a half-maximal concentration of 5.2 nM [1,4,5,6]. In addition, since cyclic guanosine-3′,5′ monophosphate (cGMP) hydrolyzes PDEs, PDE5 is cGMP specific and has obvious physiological functions such as regulation of penile erection [1]. In this aspect, AVA shows higher selectivity against PDE-6 than sildenafil and vardenafil, excellent selectivity against PDE-1 compared to sildenafil, higher selectivity against PDE11 than tadalafil, and also unique selectivity against all other PDEs [1,7].

When a literature survey was conducted for AVA, it was revealed that a limited number of analytical methods are available for its determination, which may be divided into three main groups: single-compound HPLC analysis of AVA in blood samples [3] and pharmaceutical dosage forms [8,9]; analysis of AVA with dapoxetine in binary mixtures using UV spectrophotometry [10] and HPLC [11]; and LC-MS/MS analysis of AVA in human plasma [4] and in binary mixtures containing some other acetildenafils and sildenafils [12]. Unfortunately, some of the LC methods that were described in some of the cited papers above are insufficiently validated and not tested for versatility using different instrumental configurations; besides, due to the low amount of work, the topic is still suitable to research for better identification of some analytical properties. On the other hand, there is not an official method in any of the official pharmacopoeias. In accordance with the deficiencies in the literature, the presented study was realized with a main purpose to develop robust, versatile, selective, sensitive and validated LC methods, whose analysis time, cost and efficiency are optimized for use in routine analysis of AVA in tablet formulations. The major advantages of the proposed methods were use of sophisticated analytical instrumentation and equipment to match and fulfill the recent requirements of the pharmaceutical industry standards: the use of monolithic silica columns for higher efficiency and lower analysis costs, optional utilization of either LC-DAD or LC-MS/MS instruments for quantitation, and applicability of the same chromatographic protocol for LCMS-IT-TOF, especially for extremely specific search and detection of related compounds such as impurities and degradation products in suspicious cases. Monolithic silica columns have been continuously favored since their introduction to the market, because of their low operating pressure, and higher permeability, efficiency and robustness, when compared to conventional particle columns [13,14]; in accordance, their versatility and application range was widened to cover their use in the analysis of dirty samples [15], chiral compounds [16], amino acids [17] and even viruses [18]. In accordance with today’s demands in analytical laboratories, the use of HPLC coupled with a diode array detector (LC-DAD) or mass spectrometric detection (LC-MS or LC-MS/MS) has confirmed to be the standard analytical tools for most assays used in various stages of new-drug discovery. Liquid chromatography mass spectrometry–ion trap–time of flight (LCMS-IT-TOF) is a relatively new type of mass spectrometer that combines quadrupole ion trap and time-of-flight (TOF) technologies; this results in efficient introduction of molecular ions into the ion trap and simultaneous ejection of trapped ions into the TOF region, with a continuous high-resolution MS^n^ capability on a selected fragment. LCMS-IT-TOF is widely in use to identify the compounds in complex matrices without isolating individual compounds [19,20]. High-resolution mass accuracy estimation (four digits after decimal point) can be practically realized using LCMS-IT-TOF, which makes it one of the most powerful tools for structural characterization of unknown compounds and fragment ion assignments in the product-ion spectral data.

The methods presented in this study are capable of detection of the degradation products, while they are easy to apply on pharmaceutical products of AVA on the market; the quantitation methods were applied on real samples (AVANA-50^®^ and TOP AVANA^®^ from Sunrise Remedies, Ahmedabad, India), and validated according to the International Conference on Harmonization Q2(R1) [21] and the United States Pharmacopeia (USP) [22] recommendations: Since the developed methods are stability indicating, there is no issue regarding the selectivity and specificity [21,23,24]. In addition, the degradation products were produced in forced degradation studies, qualitatively determined and accurately identified with LCMS-IT-TOF, and a novel degradation product, which was produced in oxidizing conditions, was also identified for the first time.

## 2. Results and Discussion

### 2.1. LC-DAD Method

The method development studies were initially started by searching for a universal solvent environment that could be used in all experiments and tests. AVA has two pKa values, which are predicted as 11.84 (acidic) and 5.89 (basic) [11,24]. Its log-P value is 1.84 [24], and in accordance, it has very low solubility in water, methanol and ethanol (<1 mg/mL at 25 °C), and is highly soluble in DMSO (97 mg/mL at 25 °C) [3]. On the other hand, solubility testing conducted under various pH conditions demonstrated increased solubility of AVA in acidic (pH~4) media, and decreased solubility in neutral and alkaline conditions [5], as in accordance with its log-S value of 4.2. Therefore, AVA was dissolved in ACN:DMSO mixture (94:6 *v*/*v*) for all experiments, including degradation studies.

One of the sub-purposes of the study presented in this paper was to develop qualified chromatographic methods in which very similar instrumental conditions can be applied by just changing the detector; this was considered as the key point for versatility. Accordingly, acceptable chromatographic retention with interference-free separation and determination was aimed for AVA. In order to achieve this aim, some common chromatographic starting points were evaluated and tested in the method development process. Use of an ion-suppressing agent such as acetic acid, ammonium formate or formic acid was tested at various concentrations; use of 0.1% formic acid in the mobile phase was found to achieve acceptable peak shape and ionization of AVA. Simultaneously, acetonitrile and methanol were initially tested as the organic components in the mobile phase; among these, acetonitrile provided better results. Acetonitrile has low viscosity and low absorption in the UV region; it enables better mass transfer and performs better in the solubility problem of AVA. After primary investigations, the mobile phase consisting of 0.1% formic acid in water and 0.1% formic acid in acetonitrile (75:25 *v*/*v*) came forward as the best choice for AVA. In addition to mobile phase-associated factors, use of an efficient and robust analytical column, which can also allow high-throughput separations, was preferred to increase total versatility of the methods. From this point of view, a second-generation C_18_ bonded monolithic silica column, Chromolith^®^ High-Resolution RP-18 (100 × 4.6 mm) from Merck was utilized. As a valuable advantage of the bimodal, highly porous structure of the monolithic silica skeleton, the backpressure did not exceed 36 bars in all analyses; the column temperature was set at 40 °C to increase efficiency and decrease viscosity. Furthermore, 0.5 mL/min was found as the best suitable flow rate for both LC-DAD and LC-MS/MS techniques; although the LC-DAD method allowed higher flow rates, 0.5 mL/min was the highest possible flow rate for introduction of AVA and the mobile phase into the MS/MS detector. The injection volume was preferred to be 1.0 μL for LC-DAD. Under optimized conditions, AVA was detected at about 11.8 min after injection (Supplementary Figure S1).

As well as the chromatographic parameters, detection parameters were also investigated for LC–DAD analyses. The absorptivity characteristics of AVA under the above-mentioned mobile phase conditions were monitored in the UV and visible region by using a photodiode array detector, and 247 nm was found as the highest absorbing wavelength. Since the flow rate was not high, 40 Hz data-sampling frequency was found as adequate, and detector sampling rate and time constant were set at 640 ms, which allowed at least 20 points for a chromatographic peak.

### 2.2. LC-MS/MS Method

After fine-tuning of LC parameters in the LC-DAD method development, MS/MS method development was carried out. The LC-MS/MS method was favorable for especially quantitative analysis of AVA and identification of the degradation products. Transfer of LC conditions to LC-MS/MS resulted in detection of AVA at about 12.6 min (Supplementary Figure S2); all of the LC parameters were the same except injection volume, which was 0.3 μL since very high signal intensity was observed when using MS/MS detection. The ESI source was operated in positive ion mode because no ionization was observed in negative mode. Q1 potential, collision energy (CE) and Q3 potential were the parameters optimized for multiple-reaction monitoring (MRM) transitions (Table 1). Three MRM transitions were chosen, and the dwell time of 100 ms was applied.

### 2.3. LCMS-IT-TOF Method and Identification of Forced Degradation Products

After establishment of LC and MS/MS conditions, the developed method was transferred to the LCMS-IT-TOF instrument for mass-based molecular identification applications for stability and forced degradation studies. Method transfer was successfully realized, and very similar retention was observed for AVA. Identical mobile phase composition, injection volume and column were used within the LCMS-IT-TOF method, and the AVA peak was detected at about 14.7 min.

The stability of quality control solutions is a robustness parameter to be considered in the method validation [21]; as mentioned in Section 2.4, no degradation was observed in the short- and long-term stability studies, demonstrating that AVA is a stable compound under ordinary conditions. Since stress-testing stability experiments are generally performed to determine the effect of uncontrollable or accidental exposures to abnormal conditions during production, transportation or storage, AVA was exposed to harsh conditions to fully examine its degradation potential [21,23,24]. The LCMS-IT-TOF instrument utilized in these experiments came forward as a quite useful tool for identifying and elucidating the AVA-related compounds. The proposed method allowed a more highly sensitive and accurate analysis than the ones reported in previous studies.

The degradation solutions, which were prepared according to the current practice [23,25] and protocol given under Section 3.6, were analyzed by using LC-MS/MS and LCMS-IT-TOF instruments, and the chromatograms (Supplementary Figures S3–S10) and their mass spectra were inspected for known or unknown compounds. As a result of high sensitivity of the ion-trap time-of-flight mass spectrometer, evidence of a novel and previously unknown degradation product, which was originated from oxidizing conditions, came to the forefront. It should be underlined that none of the degradation products were detected in the blank, standard and tablet assay solutions.

**Figure 2:**
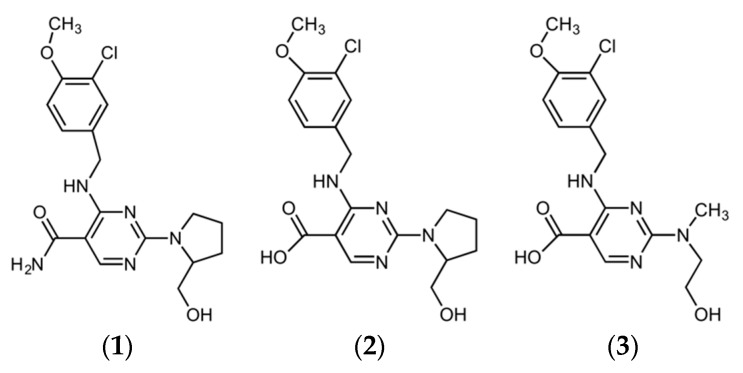
Chemical structures of the compounds detected in degradation studies: related compound **1**, related compound **2**, predicted structure of the new degradation product **3**.

Notably, related compound **1**, whose precursor ion had a *m/z* of 392, was accurately distinguished in peroxide, acid, alkali and thermal degradation conditions. In addition, related compound **2**, with *m/z* 393 as precursor ion, was observed clearly in acid and alkali degradation conditions. Related compound **1** is characterized by removal of 2-methylpyridine from the amine in the AVA molecule under various stress conditions. As a general organic chemistry approach, due to electron-withdrawing properties of the amide moiety, the bond between C and N atoms weakens, and in accordance, bond breaking can easily occur under various stress conditions. On the other hand, related compound **2** is characterized by replacement of the amide group with an acidic group (i.e., amide hydrolysis) in AVA; this type of degradation is a well-known hydrolysis reaction, which can easily occur in a variety of conditions. On the basis of high-resolution recordings of the LCMS-IT-TOF instrument, a new degradation product, whose precursor ion had a *m/z* of 366 and molecular weight predicted to be 367.1176 g/mole, was identified. The structures of the identified and predicted compounds are given in Figure 2.

There is plenty of evidence that proves the predicted molecular structure of the new compound. The peak pattern observed in the mass spectrum gives the evidence of a chlorine atom in the structure. In addition, both positive and negative ionizations of the compound give the evidence of the carboxylic acid, which normally does not exist in the structure. On the other hand, alpha-cleavage in the opening of the pyrrolidine ring, the double-bond equivalency, number of possible atoms and measured molecular weight guided the decision to the predicted molecular structure (Supplementary Figure S11).

The chromatographic and mass spectral parameters, for example, retention times, measured and predicted mass values and so on, are given in Table 2 and Supplementary Figure S12.

### 2.4. Method Validation Studies

A comprehensive analytical method validation was carried out according to ICH’s Q2 (R1) guideline to assess the reliability and trueness of the methods for the determination of AVA in pharmaceutical tablets. In accordance, results of specificity, linearity, precision, accuracy, stability and recovery tests were statistically evaluated, and LC-DAD and LC-MS/MS methods were compared with each other.

Specificity and selectivity are considered to be the most important parameters in analytical methods, because other methodological parameters or results are unreliable unless they are enabled. Realization of forced degradation studies was regarded as an official way of investigating specificity [21,23]. Degradation products are generally expected to occur in time or as a result of unsuitable storage of final products; on this basis, such compounds were produced in forced degradation studies, and discrimination of AVA and other compounds’ signals was investigated in all methods. On the other hand, selectivity of AVA in the presence of dapoxetine was an important point to clarify when combination tablets were analyzed. Since MRM was applied in LC-MS/MS and LCMS-IT-TOF methods, specificity was not regarded as a vital issue: Highly specific and structure-based mass detection overrides any other source of signal, and maintains required specificity. LC-DAD signals are more prone to influence from interfering compounds; however, since resolution between AVA and degradation products is maintained, it was accepted that specificity was achieved. In supplementary studies, which were conducted to investigate selectivity between AVA and dapoxetine, it was demonstrated that chromatographic retention behavior and detectability of dapoxetine are much different than those of AVA; an interfering dapoxetine signal was not observed in any of the assay chromatograms (Supplementary Figure S13).

It can be safely concluded that the method transfer between LC-DAD and LC-MS/MS instruments was successfully performed with minor differentiations in system suitability tests. System suitability results are shown in Table 3.

The peak area of AVA was used as the analytical signal in all of the experiments, and a plot of area versus AVA concentration was created for quantitative determination. The correlation coefficient of the calibration curve pointed out the linearity of the LC-DAD and LC-MS/MS methods over the studied concentration ranges. In addition, the precision results demonstrated that the proposed method has good performance over the tested concentration range, as summarized Table 4.

In accordance with the ICH Q (2) R1 guideline, the described methods were tested for accuracy in the range as mentioned above. Standard addition was preferred in recovery experiments, in which six independent determinations over three different concentrations were performed, covering the whole range for both methods. Data of accuracy studies are summarized in Table 5.

The robustness of an analytical method is defined as the measure of its capacity to remain unaffected by small but effective deliberate changes in method parameters. While testing the robustness of the methods by changing the mobile phase composition, pH, flow rate, column temperature and detector wavelength, the difference in retention time, number of theoretical plates and tailing-factor parameters were evaluated.

The robustness studies indicate that the retention times observed in LC-DAD and LC-MS/MS methods were more prone to deviate as a result of a 10% change in flow rate and percentage of organic phase, while number of theoretical plates and tailing factor were not changed by more than 5% in any of the robustness tests (Supplementary Table S1).

The stability of AVA solutions was investigated via analysis of 10 μg/mL AVA (for LC-DAD) and 3000 ng/mL (for LC-MS/MS) AVA after storage under different conditions. The obtained results are given Table 6.

### 2.5. Application on Real Samples

**Figure 3:**
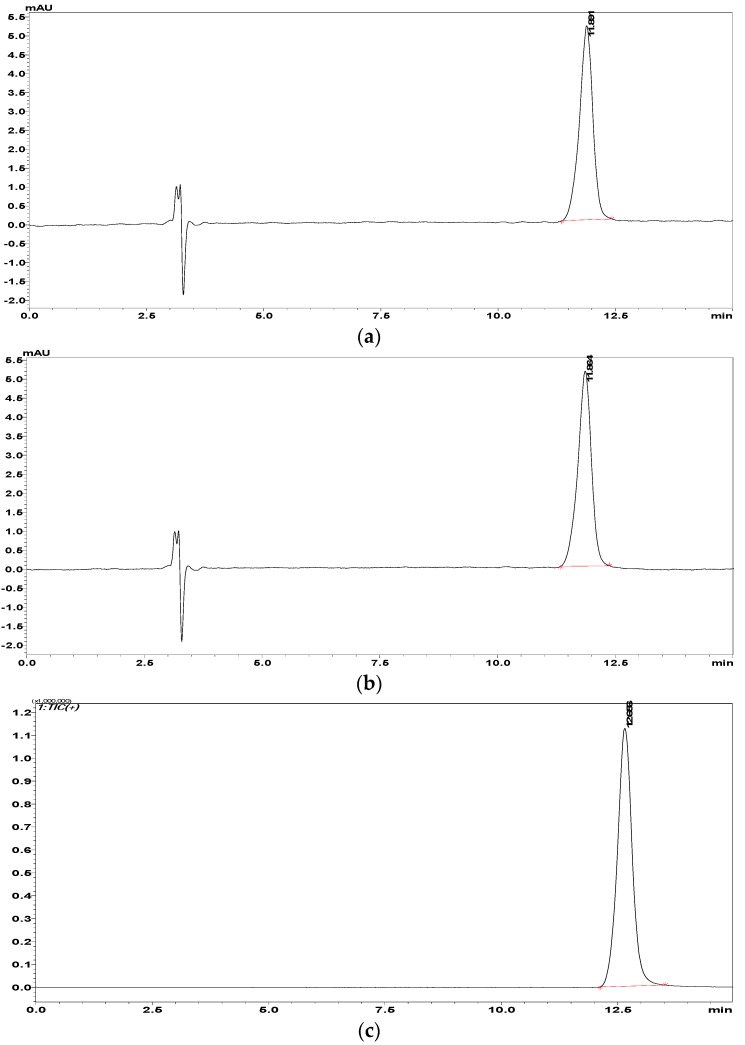

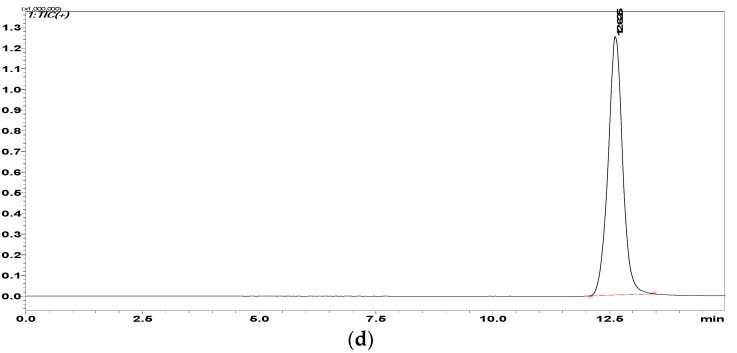
Assay chromatograms of AVANA-50^®^ (**a**) and TOP AVANA^®^ (**b**) tablet formulations for LC-DAD, and AVANA-50^®^ (**c**) and TOP AVANA^®^ (**d**) tablet formulations for LC-MS/MS.

Application of the developed methods was realized by determining AVA in real samples existing in the market. A fast dilute-and-shoot approach was employed in the current work (refer to Section 2.5). There were no interferences to AVA peaks observed in the pharmaceutical preparations of AVANA-50^®^ and TOP AVANA^®^ Film Tablet for both LC-DAD and LC-MS/MS assays. Typical chromatograms of tablet analyses are shown in Figure 3 for each of the samples and methods.

Each sample solution was injected into the system in triplicate, and average values were calculated and used as representative for quantitation. Statistical analyses regarding quantitation are given in Table 7. It can be concluded that the real samples were successfully analyzed by the validated methods, which can be accepted as good in performance from an analytical point of view.

The results of the assay studies indicated that the preparations are in accordance with the official requirements mentioned in the pharmacopoeia in terms of mass and content uniformity [26,27].

## 3. Materials and Methods

### 3.1. Chemicals and Consumables

Avanafil reference standard at 99.0% purity was purchased from Molekula GmbH (Munchen, Germany). HPLC-grade acetonitrile (ACN), formic acid (FA) and water were purchased from Sigma-Aldrich Chemie GmbH (Darmstadt, Germany); other chemicals, that is, dimethyl sulfoxide (DMSO), sodium hydroxide, hydrochloric acid and hydrogen peroxide, were analytical grade and purchased from Sigma-Aldrich Chemie GmbH (Darmstadt, Germany). All solutions were filtered through nonsterile polyvinylidene fluoride (PVDF) syringe filters (25 mm id, 0.2  μm pore size, from GS-Tek, Newark, DE, USA) prior to injection.

### 3.2. Instrumentation

Quantitative analyses were performed using a Nexera XR series liquid chromatograph, which was composed of a DGU-20A3R on-line degasser, 2 × LC-20AD gradient pumps, a SIL-20AC autosampler, a CTO-10ASVP column oven, a FCV20AH_6_ high-pressure flow line selection valve, and a CBM-20A communications bus module; flow line was changed to either LCMS-8040 triple quadrupole mass spectrometric detector for mass-based detections or SPD-M20A photodiode array detector for absorbance-based detections (all from Shimadzu, Kyoto, Japan). Also, Shimadzu LC LabSolutions 3.43 SP1 data integration software was used for instrumental control and data integration.

Qualitative analyses were performed using an LCMS-IT-TOF series hybrid ion-trap time-of-flight mass spectrometer from Shimadzu (Kyoto, Japan); the LC part of the instrument was composed of a DGU-20A3 on-line degasser, 2 × LC-20AD gradient pumps, a SIL-20A autosampler and a CBM-20A communications bus module. An SPD-M20A photodiode array detector and an IT-TOF high-resolution mass spectrometric detector was connected in series for compound identification. Instrumental control and data integration in LCMS-IT-TOF analyses were realized using LCMS Ver.3.81.418 software.

The analytical column utilized in the separations was Chromolith^®^ High Resolution RP-18e (100 mm × 4.6 mm id) from Merck KGaA (Darmstadt, Germany).

In addition, an XP-205 model analytical balance from Mettler-Toledo (Columbus, OH, USA), an RK-100 H model ultrasonic bath from Bandelin (Berlin, Germany) and a Reax-Top model vortex from Heidolph (Schwabach, Germany) were used in the preparation of samples and solutions.

### 3.3. Instrumental Parameters

The mobile phase consisted of 0.1% formic acid in water and 0.1% formic acid in acetonitrile (75:25 *v*/*v*, pH at 2.6), and pumped at the rate of 0.5 mL/min. The column oven and autosampler thermostat were set 40.0 ± 0.1 °C and 15 ± 0.1 °C, respectively. Injected sample volume was 1.0 μL for LC-DAD, and 0.3 μL for LC-MS/MS and LCMS-IT-TOF analyses.

In LC-DAD analyses, the photodiode array detector was set at 247 nm wavelength and real-time spectra were recorded between 190 and 380 nm at 40 Hz data-sampling frequency. Detector sampling rate and detector time constant were set at 640 ms.

In LC-MS/MS analyses, the detector was operated within a mass range from *m/z* 100 to *m/z* 800, using an electrospray ionization in positive ion mode (ESI+). The MS conditions were optimized as follows: drying gas (N_2_) flow was 15 L/min, nebulizing gas (N_2_) flow was 3.0 L/min, collision gas was Ar, CDL temperature was 250 °C and heat block temperature was 450 °C. Multiple reaction monitoring (MRM) mode was used throughout analyses.

LCMS-IT-TOF instrument was operated in positive ion mode with the following parameters: high-voltage probe: −3.5 kV; nebulizing gas flow: 1.5 L/min; CDL temperature: 200 °C; heat block temperature: 200 °C; drying gas pressure: 200 KPa. Collision-induced fragmentation (CID) parameters were settled as 50% for CID energy, 50% for collision gas, and Ar gas was used for CID. Detector voltage of TOF was 1.6 kV. A solution of trifluoroacetic acid was consumed as the standard sample to adjust sensitivity and resolution, and to perform mass number calibration (ion trap and TOF analyzer).

### 3.4. Preparation of Quality Control Solutions

Initial stock solution of AVA was prepared by dissolving 20.0 mg pure substance in 10.0 mL of ACN:DMSO solution (94:6 *v*/*v*); final concentration was 2000 μg/mL. Further dilutions for calibration solutions and quality control (QC) samples were prepared in acetonitrile. The solutions were found to be stable for at least 7 days when kept at 4 °C away from daylight and freeze-thaw cycles.

### 3.5. Tablet Assay Preparation

Two different tablet formulations were used to assess the applicability of the proposed methods: AVANA-50^®^ Tablets which were labelled to contain 50 mg avanafil, and TOP AVANA^®^ which was labelled to contain 50 mg avanafil and 30 mg dapoxetine (both from Sunrise Remedies, India). For the assay of each product, ten tablets were accurately weighed, the average weight of a tablet was calculated and tablets were crushed to fine particles in a mortar. The amount of a tablet powder corresponding to the average weight of a tablet (516.2 ± 4.4 mg for AVANA-50^®^ and 522.1 ± 3.7 mg for TOP AVANA^®^) was accurately weighed and transferred into a 50 mL volumetric flask. After filling up to volume with ACN:DMSO (94:6 *v*/*v*) solution, the resulting mixture solution was mechanically shaken for 5 min and sonicated for 30 min. 100 µL portion of the final mixture was transferred into a 10 mL volumetric flask, after addition of an appropriate amount of acetonitrile, it was sonicated for 10 min, made up to volume with the same solvent and filtered through 0.22 µm PVDF syringe filters before analysis.

### 3.6. Forced Degradation Studies

Forced degradation of AVA was applied under acidic, alkali, oxidative and thermal stress conditions according to ICH Q2 (R1) guidelines. Solutions were prepared by dissolving AVA in ACN:DMSO (94:6 *v*/*v*) mixture and later diluted with either distilled water, aqueous hydrochloric acid (1 M), aqueous sodium hydroxide (1 M), or aqueous hydrogen peroxide solution (30% *w*/*w*), to achieve a concentration of 10 mg/mL. The resulting solutions were kept at 80 °C for 24 h. After degradation applications, solutions were diluted with acetonitrile to obtain an expected AVA concentration of 10 μg/mL. The results were also compared with those of the stability studies.

### 3.7. Method Validation

#### 3.7.1. System Suitability Testing

As an essential part of the LC method development, system suitability was studied to interpret the chromatographic performance of the LC instrument. Resolution, tailing factor, number of theoretical plates, peak width and height equivalent to a theoretical plate were calculated via Shimadzu LC LabSolutions 5.86 SP1 data integration software.

#### 3.7.2. Specificity

In the ICH Q2(R1) guideline, utilization of a second well-characterized analytical procedure is suggested to assess the specificity of the proposed method or whether the analytical signals are distinct from possible interferences such as impurities or degradation products. Likewise, possible effects of AVA impurities, degradation products or other pharmaceutical excipients were studied, and the results were used to determine specificity. The chromatograms and peaks of interest were investigated for suspicious and uncertain signals to prove that a positive or negative response was not observed. Besides, peak purities were checked using a photodiode array and mass detector to verify that the analytical signal was not attributable to more than one compound.

#### 3.7.3. Linearity and Range

The linearity plot was examined over a number of concentration levels of AVA solutions. The solutions prepared for LC-DAD analyses were 0.500, 1.50, 2.50, 3.00, 5.00, 6.00, 7.00, 8.00, 9.00, 10.0, 11.0, 12.0, 12.5, 15.0 and 20.0 μg/mL (*n* = 15); and 150, 450, 750, 900, 1500, 1800, 2100, 2400, 2700, 3000, 3300, 3600, 4500, 5250 and 6000 ng/mL (*n* = 15) for LC-MS/MS methods. The ranges of the methods corresponded to 5–200% of predicted concentration of the test solutions. Each solution was injected in triplicate and average values were used as representative. Slope, intercept and correlation coefficient, as well as confidence intervals of the slope and the intercept at 95% confidence level, were calculated using GraphPad Prism version 6.0e statistical analysis software.

#### 3.7.4. Accuracy

Recovery studies were performed to determine accuracy. Pre-analyzed tablets (both of AVANA-50^®^ and TOP AVANA^®^) were spiked with known amounts of AVA at 3 different levels (80%, 100% and 120%), which corresponded to low (for LC-DAD, 8.00 µg/mL; for LC-MS/MS 2400 ng/mL), medium (for LC-DAD 10.0 µg/mL; for LC-MS/MS 3000 ng/mL) and high (for LC-DAD 12.0 µg/mL; for LC-MS/MS 3600 ng/mL) fortification. Three parallel sets were prepared for each level. Spiked tablet samples were re-analyzed, and mean recovery, standard deviation, RSD % and confidence limits at 95% confidence level were calculated.

#### 3.7.5. Precision

Precision experiments included intraday and interday (intermediate) studies. Precision was interpreted by analyzing reference and tablet sample solutions including 10.0 µg/mL AVA for LC-DAD and 3000 ng/mL AVA for LC-MS/MS 8 times using the recommended method on the same and 3 consecutive days. Results were statistically evaluated including mean, standard error of mean, standard deviation, RSD% and confidence interval at 95% confidence level using GraphPad Prism version 6.0e.

#### 3.7.6. Limits of Detection and Quantification

The limit of detection (LOD) and limit of quantification (LOQ) were calculated according to ICH recommendations, employing the inspection of the baseline noise (blank signal) in the chromatograms. The average noise of the whole baseline in the chromatograms was measured (N) and it was calculated as 3.3 for LOD and 10.0 for LOQ. The calculated values were regarded as the lowest concentration for detection and quantitation.

#### 3.7.7. Robustness

Robustness was investigated by analyzing 10 μg/mL AVA (For LC-DAD) and 3000 ng/mL (for LC-MS/MS) solutions. Within this context, initial values of the organic component of the mobile phase composition, pH, flow rate, column temperature and detector wavelength were changed intentionally; then, the difference observed in retention time, number of theoretical plates and tailing factor were evaluated.

#### 3.7.8. Stability

The initial stock solution of AVA (2000 μg/mL) was suitably diluted to 10 μg/mL for LC-DAD and 3000 ng/mL for LC-MS/MS. The resulting solutions were injected periodically (6, 12, 18, 24, 48 h, at −20 °C for 2 weeks and three freeze-thaw cycles) into the column, and results were compared against a blank to study the stability of AVA solutions and mobile phase.

## 4. Conclusions

In this paper; quantitative analysis of AVA in pharmaceutical preparations, qualitative analysis of AVA and its degradation products, and identification of a novel degradation product were successfully performed by using the same analytical conditions on LC-DAD, LC-MS/MS and LCMS-IT-TOF instruments. Briefly, either LC-DAD or LC-MS/MS techniques can be used for an interference-free determination of AVA as well its degradation products and dapoxetine. Both of the quantitative analysis methods were successfully validated and can be used reliably in routine assays of QC laboratories. The methods allowed detection and quantitation of AVA within 11.8 min and 12.6 min when LC-DAD and LC-MS/MS instruments were used, respectively. The most important feature of the proposed methods was their versatility, by which any of them can be preferred, or transferred easily according to the instrumental facilities of the laboratories. The proposed LC-DAD method is found to be similar in terms of LOD, LOQ, linearity and recovery performance when compared to previously reported absorbance-based methods [3,8,9,10], while the LC-MS/MS method was better than previously reported ones [3,8,9,10,11,12] in terms of sensitivity since it has lower LOD and LOQ values; in addition, both methods are better in precision. The methods are well characterized to be used for routine release and stability testing assays for AVA tablets. On the other hand, superiority of LCMS-IT-TOF was shown for investigation of new degradation products. AVA was detected at about 14.7 min, while retention times for related compounds **1** and **2**, and the new degradation product, were 13.3, 20.5 and 33.5 min, respectively; discovery of a new degradation product was one of the most important aspects of the paper. It is thought that each of the methods should significantly contribute to the field of pharmaceutical analysis and clinical and bioanalytical research with labor-, time- and money-saving advantages.

## Figures and Tables

**Table 1:** MRM conditions of avanafil in LC-MS/MS analyses.

Compound	Precursor Ion	Product Ion	Q1 Pre-Bias (V)	CE (V)	Q3 Pre-Bias (V)
		155.05	−22.0	−47.0	−27.0
Avanafil	483.95	375.10	−22.0	−28.0	−25.0
		233.10	−22.0	−36.0	−23.0

**Table 2:** Some characteristics of AVA and its degradation products obtained using the LCMS-IT-TOF instrument operated in positive ion mode.

Compounds	t_R_ (min)	Measured *m/z* [M − H]^+^	Predicted *m/z* [M − H]^+^	Error (ppm)	Isotope	λmax (nm)	Double-Bond Equivalent	Molecular Formula
Related compound **1**	13.3	392.1485	392.1484	0.26	87.74	247	10.0	C_18_H_22_ClN_5_O_3_
Avanafil	14.7	484.1857	484.1858	−0.21	80.03	247	14.0	C_23_H_26_ClN_7_O_3_
Related compound **2**	20.5	393.1320	393.1324	−1.02	91.39	245	10.0	C_18_H_21_ClN_4_O_4_
New degradant	33.5	367.1176	367.1168	2.18	86.78	243	9.00	C_16_H_19_ClN_4_O_4_ (Predicted)

**Table 3:** Results of system suitability tests for AVA determination using LC-DAD and LC-MS/MS methods (*n* = 10).

Parameter	LC-DAD	LC-MS/MS	Reference Value
Retention time ± SD (*t_R_*, min)	11.8 ± 0.05	12.6 ± 0.05	N/A
Relative standard deviation (%) of *t_R_*	0.381	0.382	RSD ≤ 1%
Injection precision (retention time) (min)	0.229	0.401	RSD ≤ 1%
Tailing factor (*T*)	0.963	0.975	T ≤ 2
Number of theoretical plates (*N*)	8089	7661	N > 2000
Resolution (*R_s_*)	17.558	N/A	>2
Peak width (*W*)	0.534	0.576	≤1
Height equivalent to a theoretical plate (*H*)	18.55	19.58	

**Table 4:** Statistical data regarding linearity and precision studies.

Parameter	LC-DAD	LC-MS/MS
Linearity range (*n* = 15)	0.5–20 μg/mL	150–6000 ng/mL
Slope ± SD (*n* = 15)	10,688 ± 89.01	8584 ± 123.4
Intercept ± SD (*n* = 15)	−2870 ± 865.7	−1.034 × 10^6^ ± 378,745
Regression coefficient (*n* = 15)	0.9991	0.9973
CI^1^ of the slope (*n* = 15)	10,496–10,880	8317–8850
Limit of quantitation	0.217 μg/mL	3.55 ng/mL
Limit of detection	0.072 μg/mL	1.17 ng/mL
Repeatability (intraday, mean ± SD, *n* = 10)	102,930 ± 585.1	2.44 × 10^7^ ± 188,093
Repeatability (intraday, RSD %, *n* = 10)	0.57	0.77
Repeatability (intraday, SEM ^2^, *n* = 10)	185.0	59,481
Repeatability (intraday, CI ^1^, *n* = 10)	±419	0.014 × 10^7^
Repeatability (interday, mean ± SD, *n* = 6 × 3 days)	102,728 ± 740.2	2.456 × 10^7^ ± 426,029
Repeatability (interday, RSD %, *n* = 6 × 3 days)	0.72	1.73
Repeatability (interday, SEM ^2^, *n* = 6 × 3 days)	174.5	100,416
Repeatability (interday, CI ^1^), *n* = 6 × 3 days)	±368	0.021 × 10^7^

^1^ Confidence interval at 95% confidence level; ^2^ standard error of mean.

**Table 5:** Statistical evaluation of accuracy studies performed.

	Added ^1^	Found ± SD ^1^	Recovery ± SD (%)	RSD (%)	Mean Recovery ± SD (%)
LC-DAD	AVANA-50^®^				
	8.000	7.8820 ± 0.2815	98.525 ± 0.2815	3.5711	97.621 ± 0.8046
	10.000	9.7356 ± 0.0356	97.356 ± 0.0356	0.3652	
	12.000	11.6379 ± 0.2210	96.983 ± 0.2210	1.8992	
	TOP AVANA^®^				
	8.000	7.5770 ± 0.2978	94.712 ± 3.7227	3.9305	95.429 ± 3.5604
	10.000	9.9293 ± 0.3640	99.293 ± 3.6405	3.6664	
	12.000	11.0738 ± 0.2114	92.2815 ± 1.7614	1.9087	
LC-MS/MS	AVANA-50^®^				
	3200	3229 ± 38	100.91 ± 1.1787	1.1681	100.82 ± 0.1560
	4000	4026 ± 10	100.64 ± 0.2421	0.2406	
	4800	4844 ± 38	100.91 ± 0.7869	0.7798	
	TOP AVANA^®^				
	3200	3265 ± 70	102.03 ± 2.1839	2.1405	102.09 ± 0.3635
	4000	4071 ± 11	101.77 ± 0.2654	0.2608	
	4800	4919 ± 134	102.49 ± 2.8450	2.7760	

^1^ The amounts are in μg/mL for LC-DAD and ng/mL for LC-MS/MS.

**Table 6:** The results of stability studies for 10 μg/mL AVA (*n* = 6).

Added	Short-Term (24 h, rt)		Medium-Term (48 h, rt)		Long-Term (2 weeks, −20 °C)		Freeze–Thaw (3 cycles)	
	Found (mean)	RSD (%)	Found (mean)	RSD (%)	Found (mean)	RSD (%)	Found (mean)	RSD (%)
10.0 (μg/mL) (LC-DAD)	9.8	0.34	9.9	0.51	9.9	0.81	9.8	0.79
3000.0 (ng/mL) (LC-MS/MS)	3114.6	0.43	3108.9	0.19	3118.6	0.16	2960.2	0.36

No degradation was observed at the end of stability studies.

**Table 7:** Assay results of AVANA-50^®^ and TOP AVANA^®^ Film Tablets (*n* = 10).

	AVANA-50^®^		TOP AVANA^®^	
Parameters	LC-DAD	LC-MS/MS	LC-DAD	LC-MS/MS
Mean ^1^ ± SD (mg)	46.21 ± 1.25	47.34 ± 1.29	47.93 ± 3.77	49.52 ± 3.06
Standard error of mean (mg)	0.40	0.41	1.12	0.97
Median (mg)	46.54	47.50	49.57	50.44
RSD ^2^ (%)	2.71%	2.73%	7.85%	6.18%
Confidence interval ^3^	45.32–47.11	46.41–48.26	45.24–50.62	47.33–51.71
*t*-test value (*p*-value)	1.975 (0.0638)		1.037 (0.3136)	
F-test value (*p*-value)	1.067 (0.9250)		1.510 (0.5493)	

^1^ Amount of AVA per tablet. ^2^ RSD: relative standard deviation. ^3^ Confidence interval at 95% confidence level (mg).

## Supplementary Materials

The supplementary materials are available online. Figure S1: LC-DAD chromatogram of a standard AVA solution (C = 10 µg/mL) recorded under optimized conditions, Figure S2: Total ion chromatogram of a standard AVA solution (C = 3000 ng/mL) recorded under optimized LC-MS/MS conditions, Figure S3–S10: DAD (a) and MS (b) chromatograms of degradation samples and corresponding blanks recorded using LCMS-IT-TOF instrument, Figure S11: Possible production pathway of the new degradation product, Figure S12: LCMS-IT-TOF high-resolution mass spectra of AVA and degradation products, Figure S13: Assay TIC (+) chromatogram of TOP AVANA^®^ recorded using LC-MS/MS instrument. Table S1: Robustness results for LC-DAD and LC-MS/MS methods.
