# Supplementary material for: Development of Validated and Stability-Indicating LC-DAD and LC-MS/MS Methods for Determination of Avanafil in Pharmaceutical Preparations and Identification of a Novel Degradation Product by LCMS-IT-TOF
Source: Molecules. 2018 Jul 19;23(7):1771. doi: 10.3390/molecules23071771 (PMC6100578; doi:10.3390/molecules23071771)
Supplement: Supplementary file 1 [file molecules-23-01771-s001.pdf]

Table S1. Robustness results for LC-DAD and LC-MS/MS methods (n=3 for each condition).

Parameter		Retention time (min)	Diff. [1] (%) ± SD	Number of theoretical plates ± SD	Diff. [1] (%) ± SD	Tailing factor ± SD	Diff. [1] (%) ± SD
LC-DAD		11.83 ± 0.05		8851 ± 40		0.91 ± 0.02	
Flow rate (mL/min)	0.45	13.22 ± 0.03	11.43 ± 0.24	8828 ± 96	0.74 ± 0.67	0.94 ± 0.003	2.98 ± 0.34
	0.55	10.98 ± 0.03	7.41 ± 0.22	8533 ± 25	3.59 ± 0.28	0.91 ± 0.03	2.68 ± 0.42
Column temperature (°C)	35	12.19 ± 0.02	3.99 ± 2.36	8803 ± 71	0.79 ± 0.37	0.92 ± 0.01	1.19 ± 0.58
	45	11.90 ± 0.03	0.29 ± 0.21	8533 ± 72	3.59 ± 0.81	0.94 ± 0.03	3.18 ± 2.99
Percentage of organic phase (%)	22.5	19.13 ± 0.04	61.24 ± 0.30	8779 ± 179	1.71 ± 0.84	0.95 ± 0.04	4.00 ± 4.55
	27.5	8.27 ± 0.05	30.27 ± 0.40	8418 ± 98	4.89 ± 1.10	0.92 ± 0.01	1.53 ± 1.03
Detector wavelength (nm)	245	12.03 ± 0.02	1.43 ± 0.15	8494 ± 23	4.04 ± 0.25	0.91 ± 0.07	0.48 ± 0.33
	249	12.03 ± 0.02	1.42 ± 0.15	8491 ± 43	4.07 ± 0.48	0.92 ± 0.01	0.65 ± 0.37
LC-MS\MS		12.63 ± 0.05		8401 ± 154		0.97 ± 0.01	
Flow rate (mL/min)	0.45	13.82 ± 0.03	9.39 ± 0.19	8170 ± 81	2.76 ± 0.97	0.97 ± 0.01	0.84 ± 0.33
	0.55	11.56 ± 0.04	8.48 ± 0.29	8342 ± 114	1.27 ± 0.44	0.97 ± 0.01	0.74 ± 0.49
Column temperature (°C)	35	12.78 ± 0.02	1.15 ± 0.14	7987 ± 109	4.93 ± 1.29	0.99 ± 0.01	2.24 ± 1.40
	45	12.49 ± 0.02	1.18 ± 0.16	7993 ± 226	5.17 ± 2.69	0,97 ± 0.05	0.57 ± 0.47
Percentage of organic phase (%)	22.5	19.72 ± 0.05	56.12 ± 0.38	8108 ± 341	3.94 ± 3.39	0.97 ± 0.01	1.35 ± 4.39
	27.5	8.86 ± 0.05	29.90 ± 0.38	7974 ± 129	5.09 ± 1.53	1.00 ± 0.01	3.16 ± 0.62

[1] Dif.: Difference

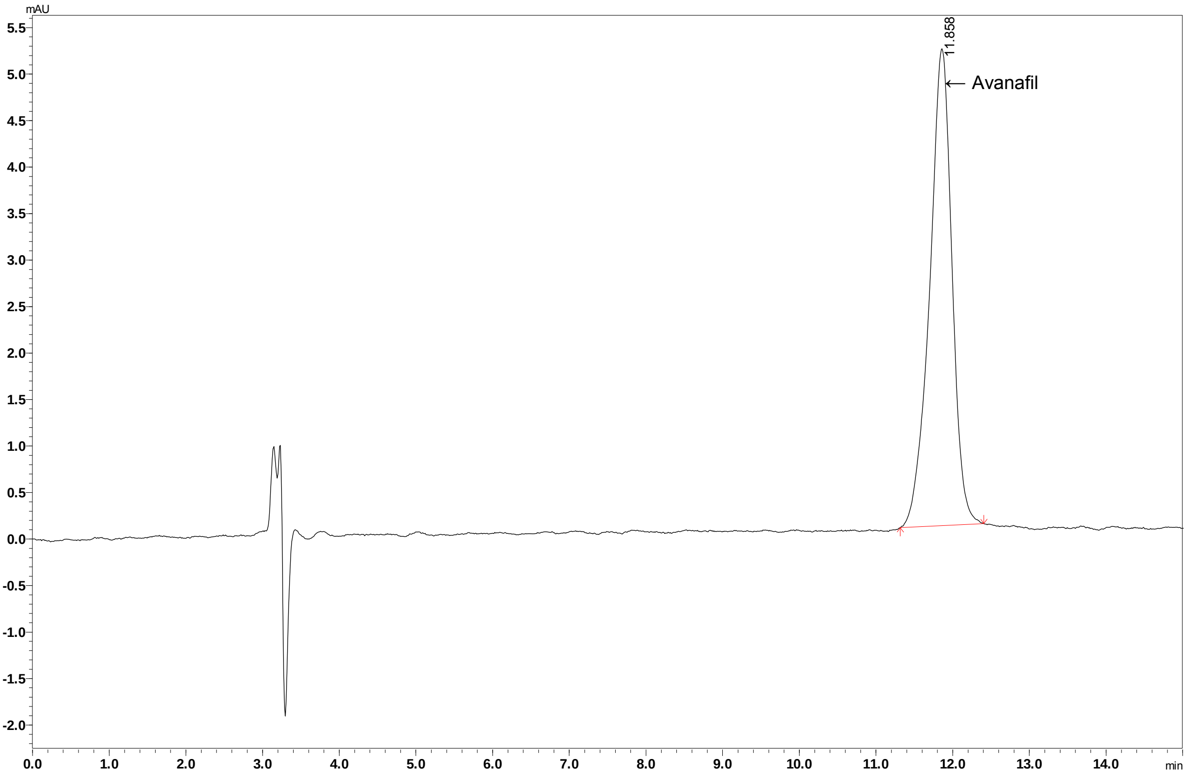

Figure S1. LC-DAD chromatogram of a standard AVA solution (C=10 μg/mL) recorded under optimized conditions.

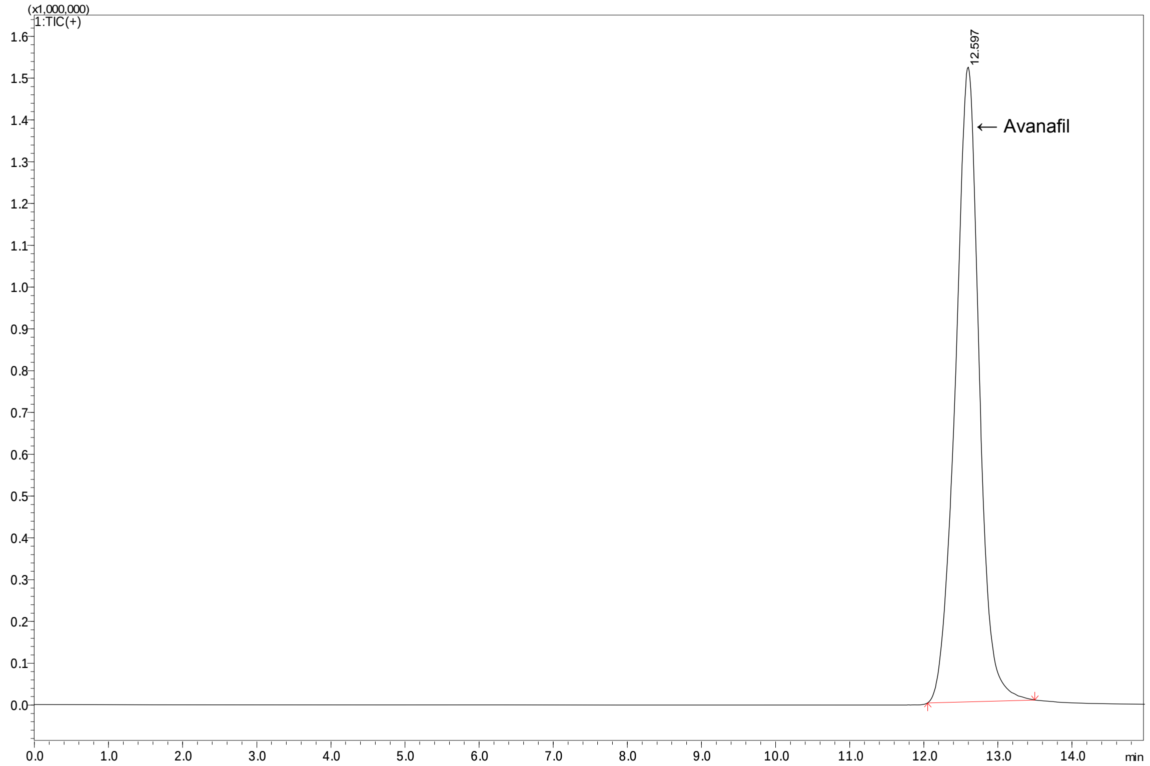

Figure S2. Total ion chromatogram of a standard AVA solution (C=3000 ng/mL) recorded under optimized LC-MS/MS conditions.

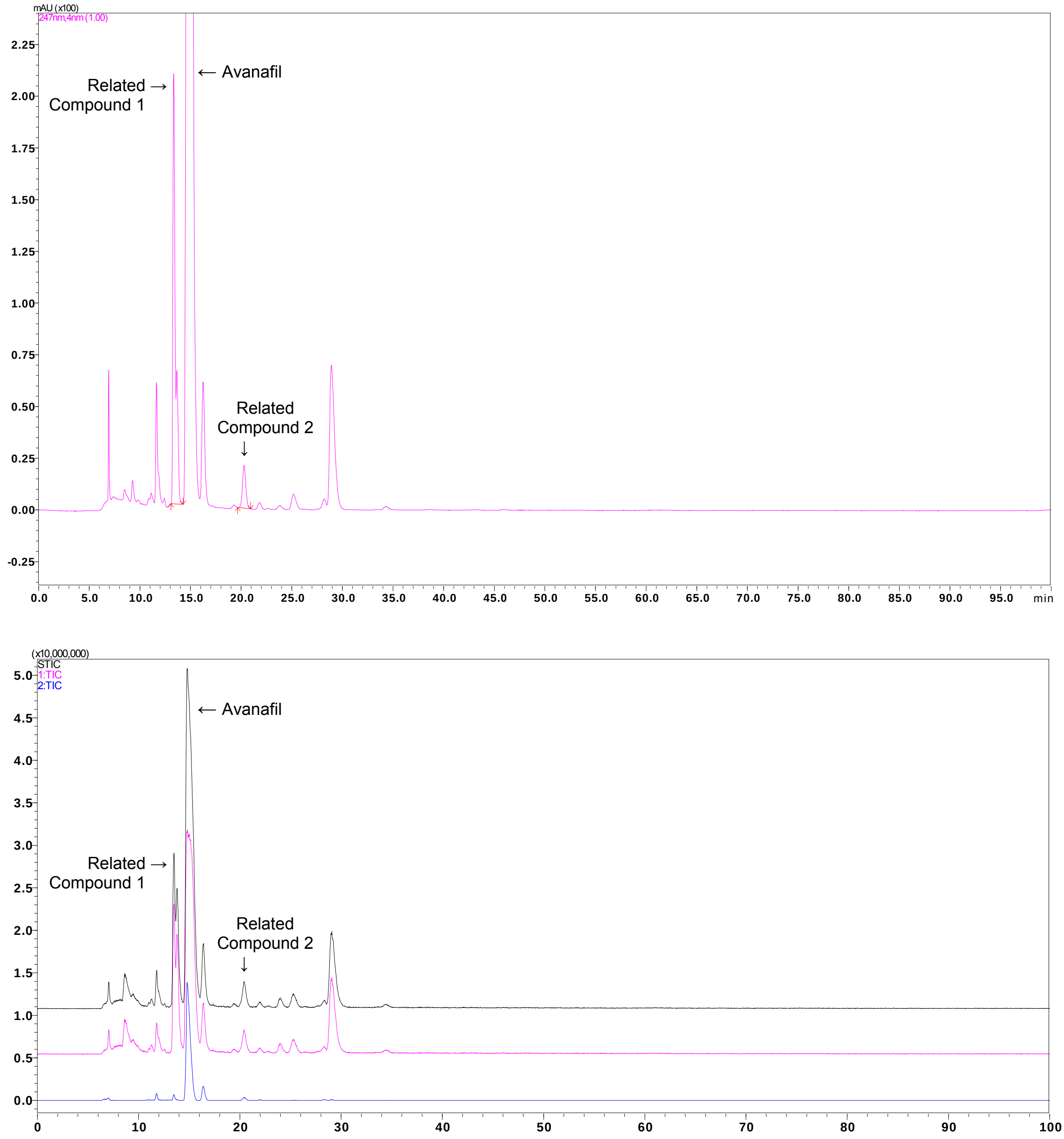

Figure S3. DAD (a) and MS (b) chromatograms of an acidic degradation sample recorded using LCMS-IT-TOF instrument (STIC: Sum of total ion chromatograms, 1:TIC: Total ion chromatogram recorded in positive ion mode, 2:TIC: Total ion chromatogram recorded in negative ion mode).

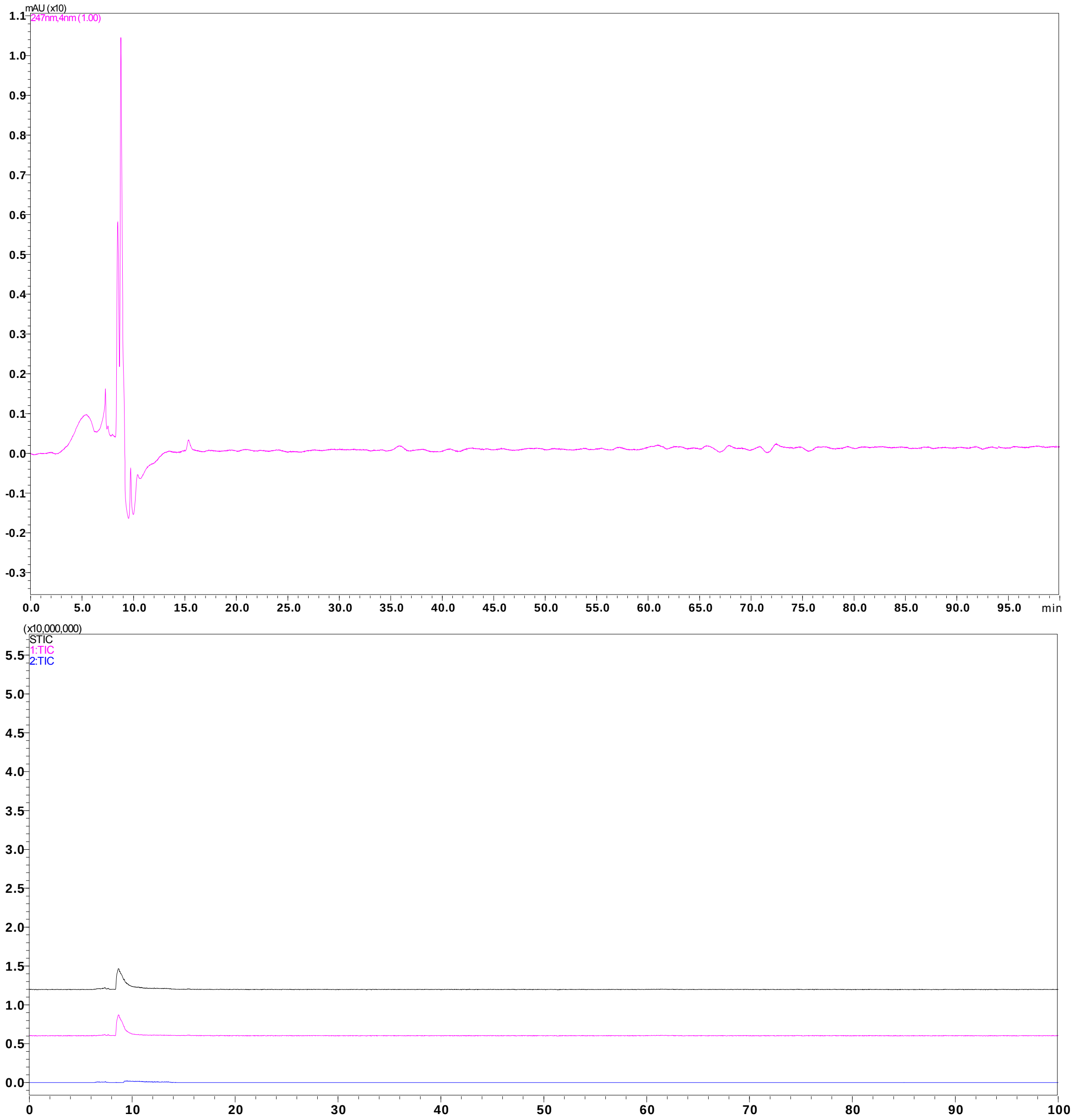

Figure S4. DAD (a) and MS (b) chromatograms of a blank solution used in acidic degradation experiments recorded using LCMS-IT-TOF instrument (STIC: Sum of total ion chromatograms, 1:TIC: Total ion chromatogram recorded in positive ion mode, 2:TIC: Total ion chromatogram recorded in negative ion mode).

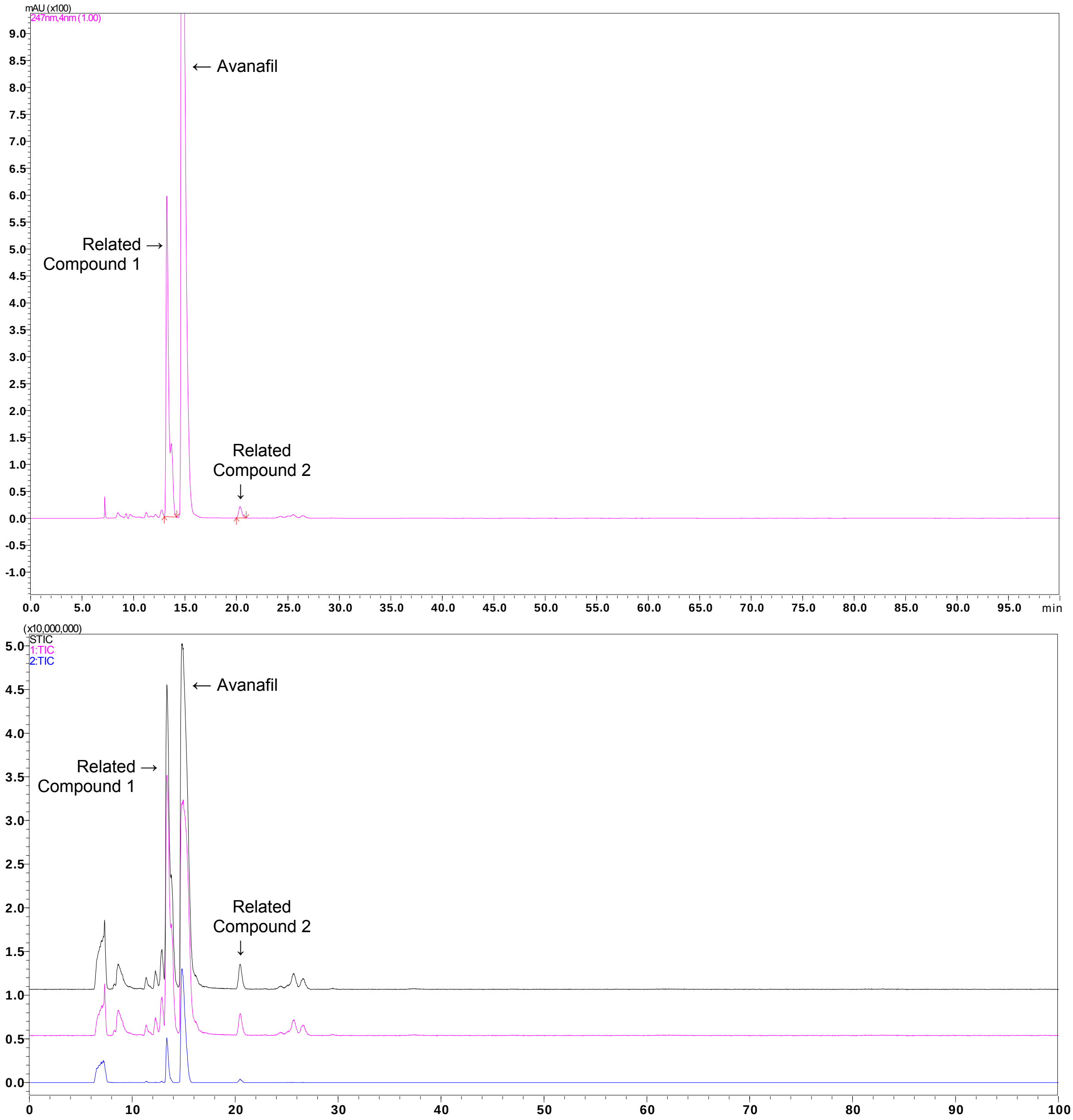

Figure S5. DAD (a) and MS (b) chromatograms of an alkali degradation sample recorded using LCMS-IT-TOF instrument (STIC: Sum of total ion chromatograms, 1:TIC: Total ion chromatogram recorded in positive ion mode, 2:TIC: Total ion chromatogram recorded in negative ion mode).

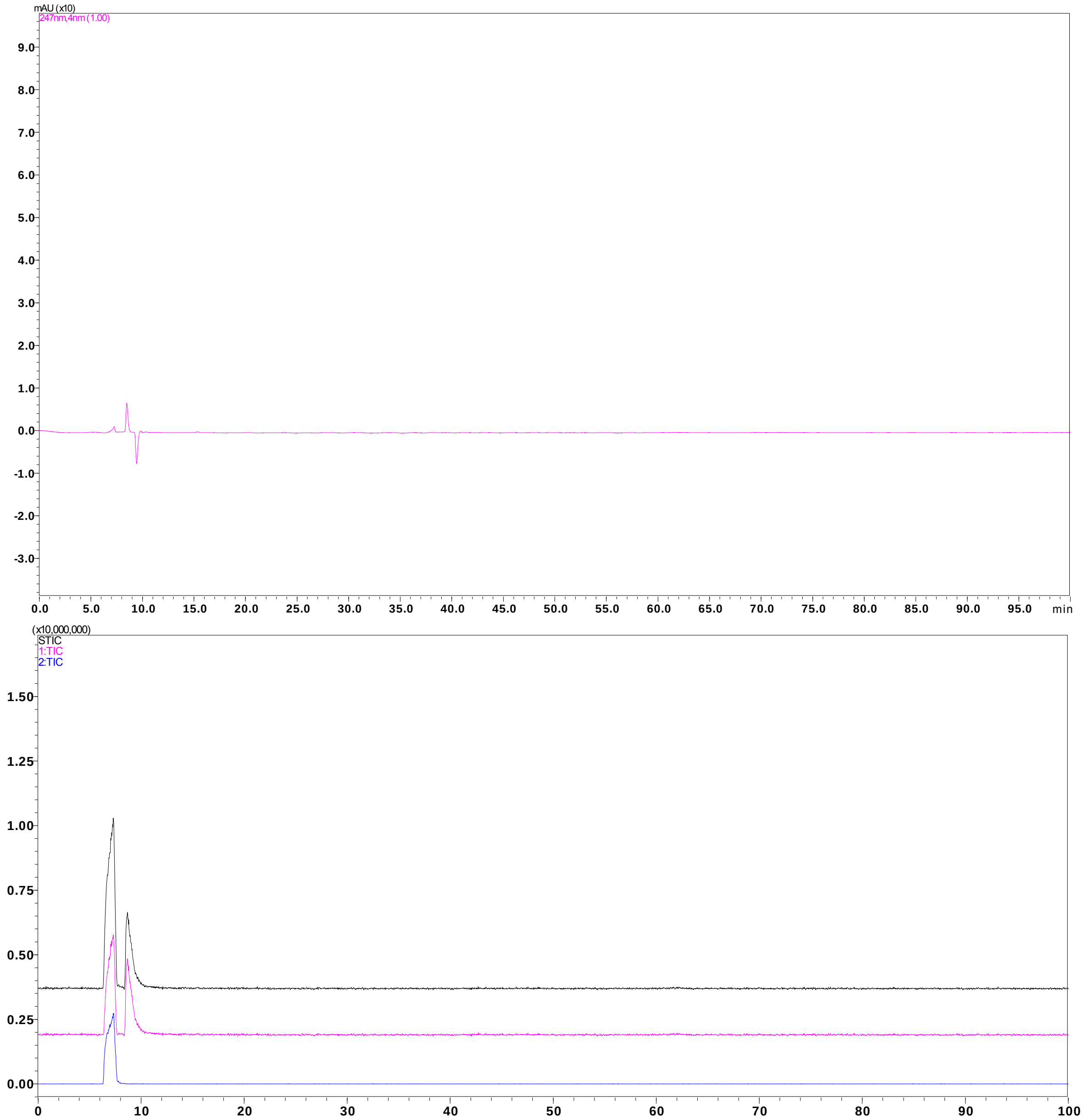

Figure S6. DAD (a) and MS (b) chromatograms of a blank solution used in alkali degradation experiments recorded using LCMS-IT-TOF instrument (STIC: Sum of total ion chromatograms, 1:TIC: Total ion chromatogram recorded in positive ion mode, 2:TIC: Total ion chromatogram recorded in negative ion mode).

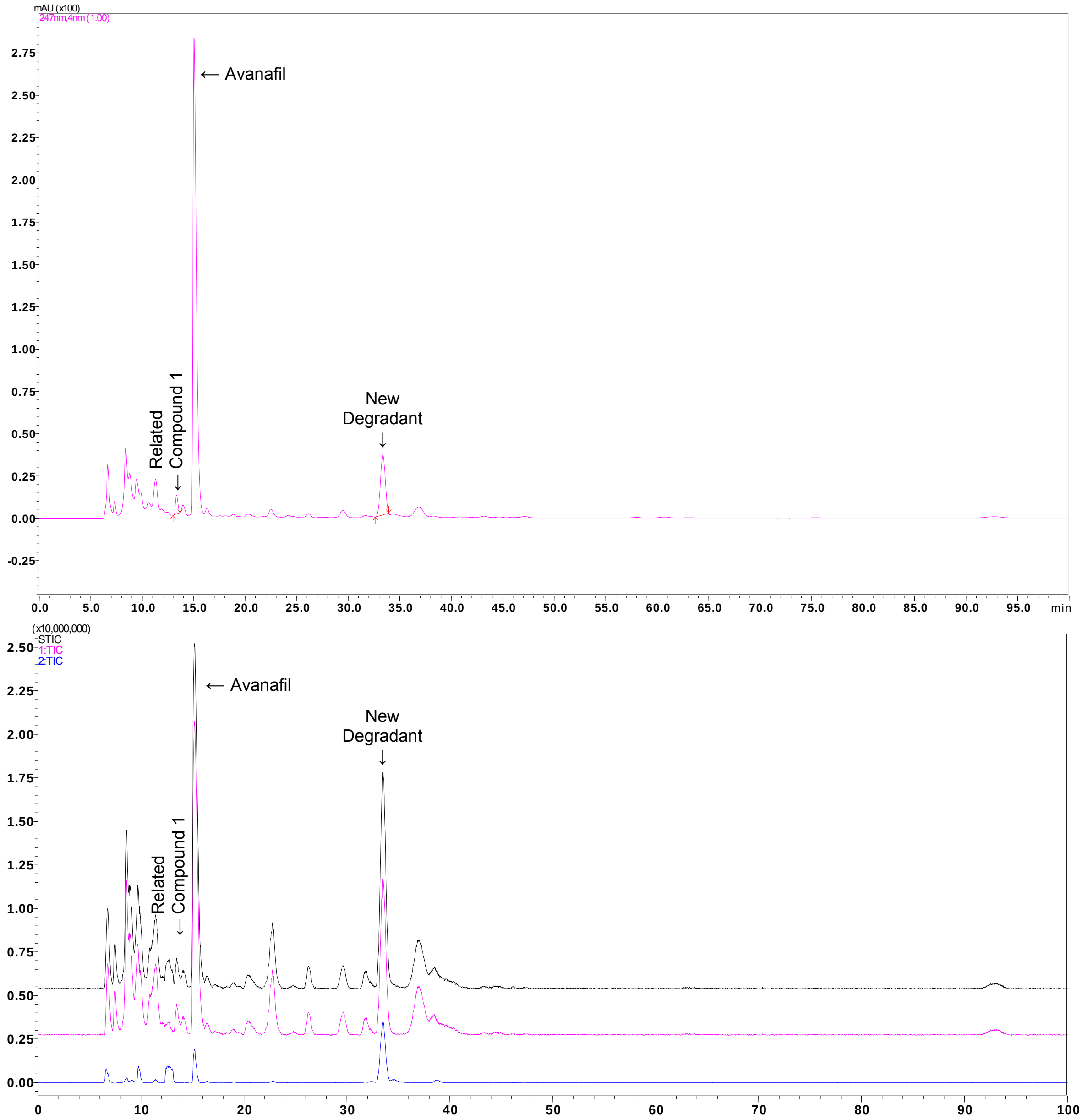

Figure S7. DAD (a) and MS (b) chromatograms of an oxidative degradation sample recorded using LCMS-IT-TOF instrument (STIC: Sum of total ion chromatograms, 1:TIC: Total ion chromatogram recorded in positive ion mode, 2:TIC: Total ion chromatogram recorded in negative ion mode).

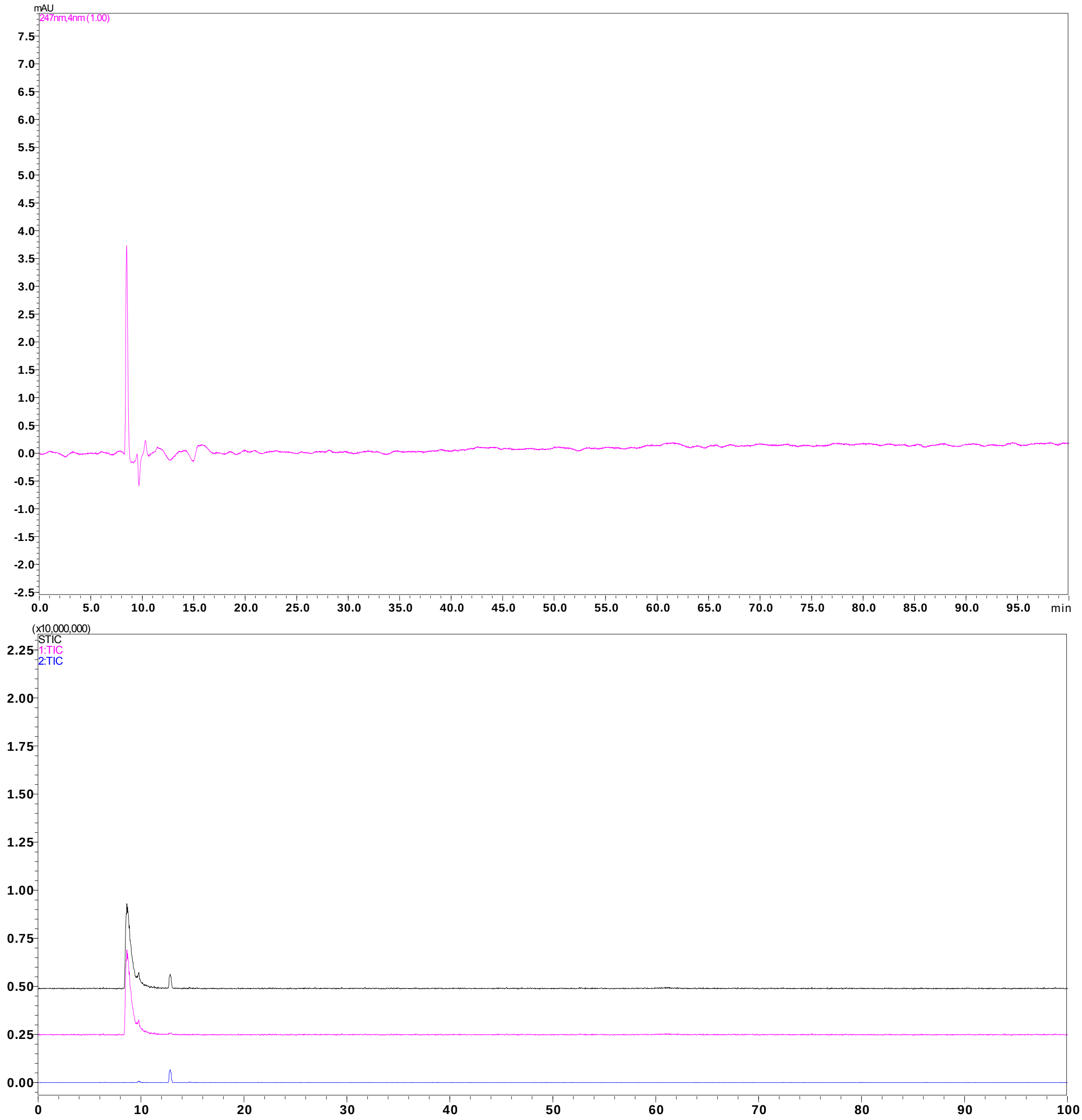

Figure S8. DAD (a) and MS (b) chromatograms of a blank solution used in oxidative degradation experiments recorded using LCMS-IT-TOF instrument (STIC: Sum of total ion chromatograms, 1:TIC: Total ion chromatogram recorded in positive ion mode, 2:TIC: Total ion chromatogram recorded in negative ion mode).

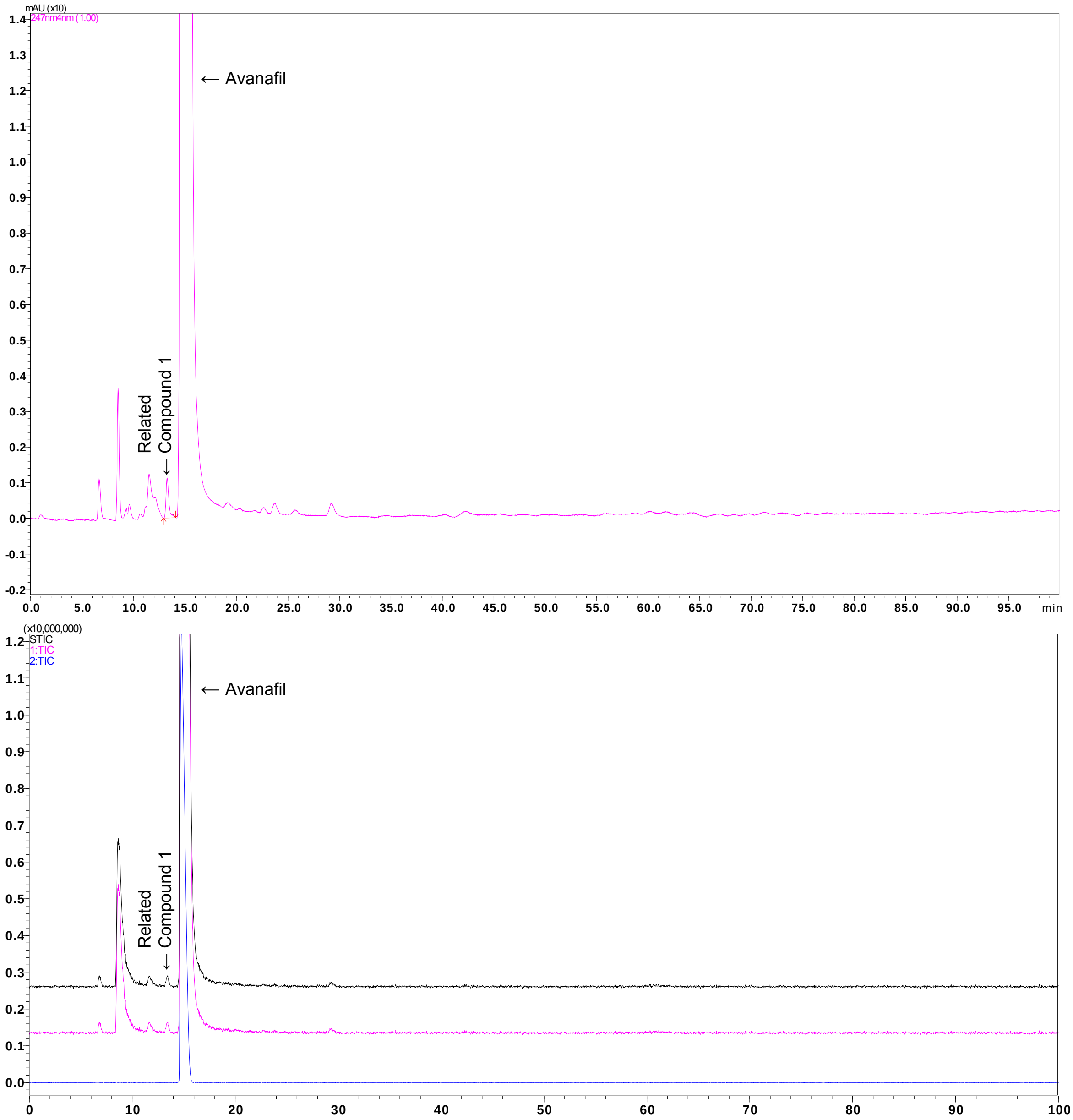

Figure S9. DAD (a) and MS (b) chromatograms of a heat-degradation sample recorded using LCMS-IT-TOF instrument (STIC: Sum of total ion chromatograms, 1:TIC: Total ion chromatogram recorded in positive ion mode, 2:TIC: Total ion chromatogram recorded in negative ion mode).

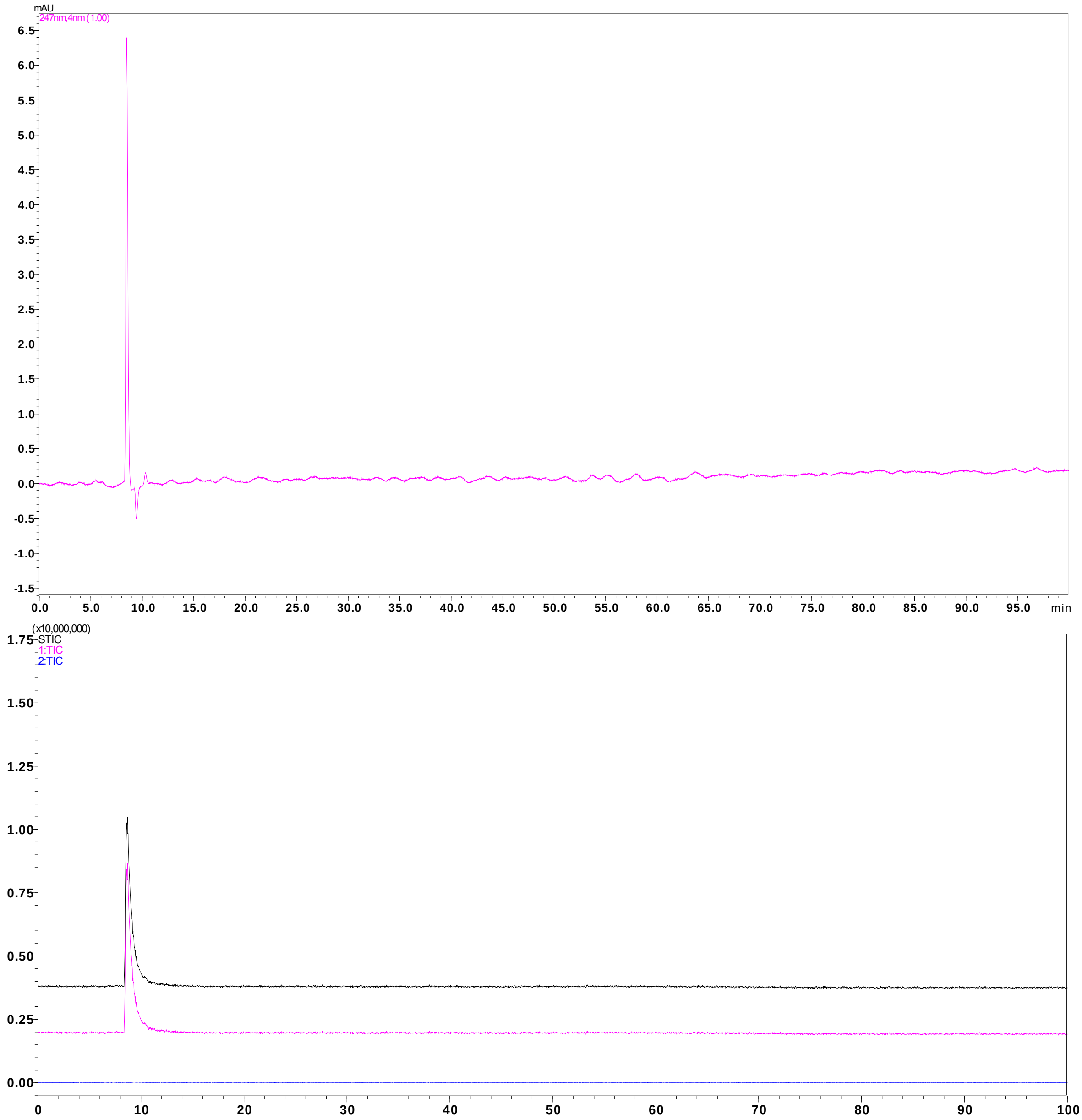

Figure S10. DAD (a) and MS (b) chromatograms of a blank solution used in heat-degradation experiments recorded using LCMS-IT-TOF instrument (STIC: Sum of total ion chromatograms, 1:TIC: Total ion chromatogram recorded in positive ion mode, 2:TIC: Total ion chromatogram recorded in negative ion mode).

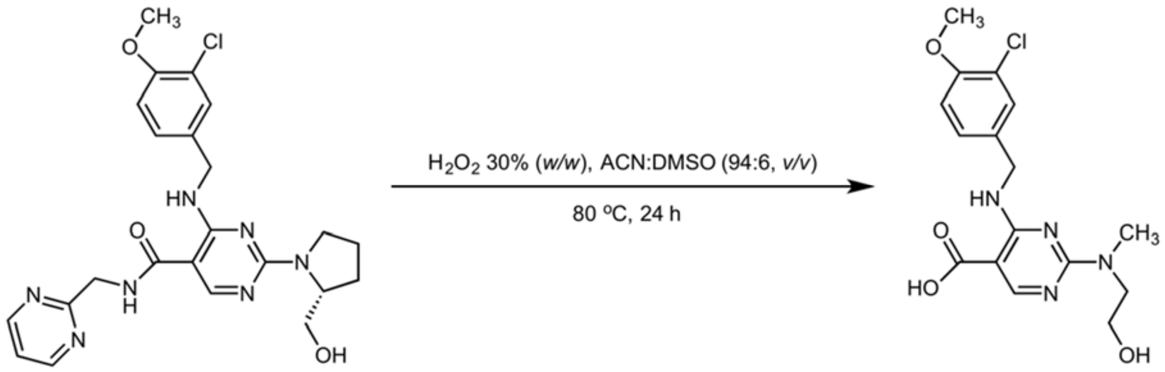

Figure S11. Possible production pathway of the new degradation product.

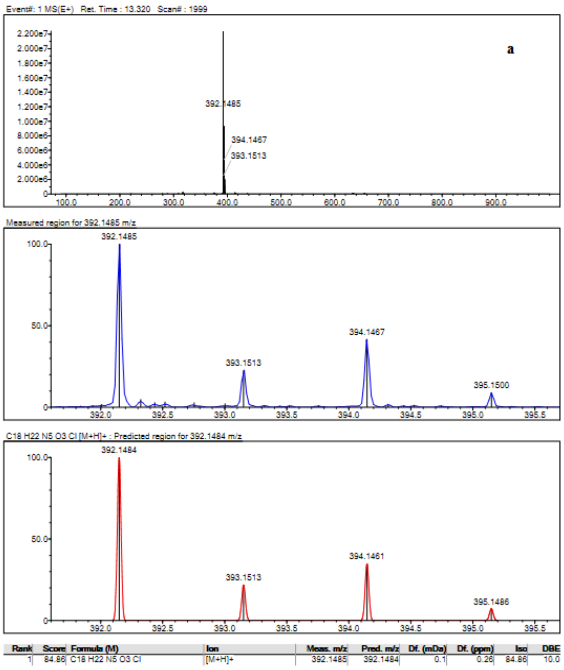

(**a**)

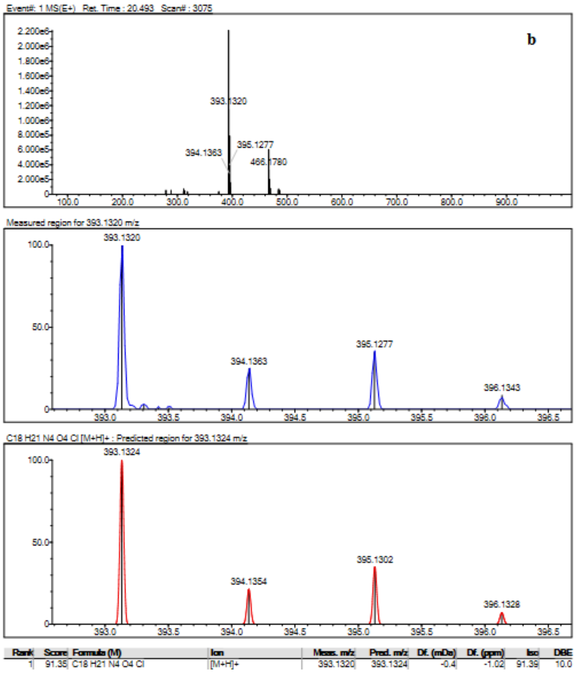

(**b**)

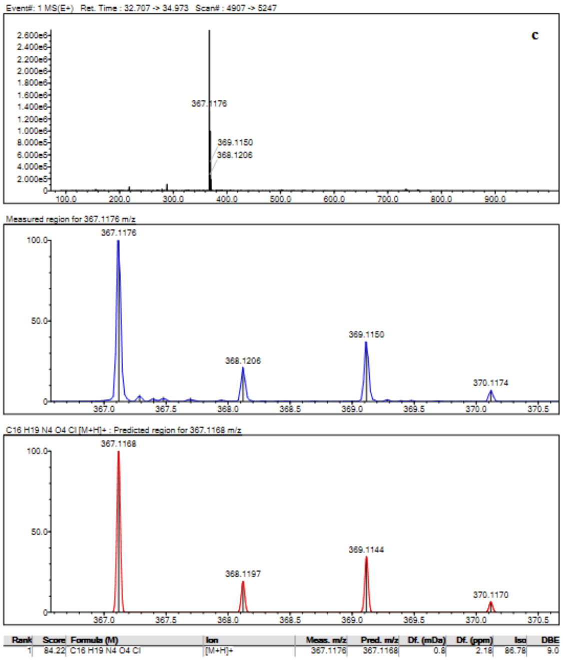

(**c**)

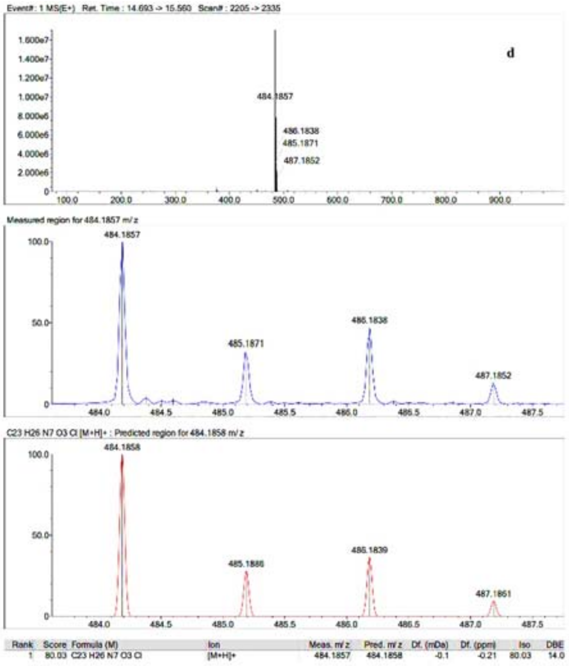

(**d**)

Figure S12. LCMS-IT-TOF high-resolution mass spectra of AVA and degradation products: Alkali degradation spectrum of related compound 1 (**a**), acidic degradation spectrum of related compound 2 (**b**) and peroxide degradation spectrum of the newly identified compound (**c**) and AVA spectrum (**d**).

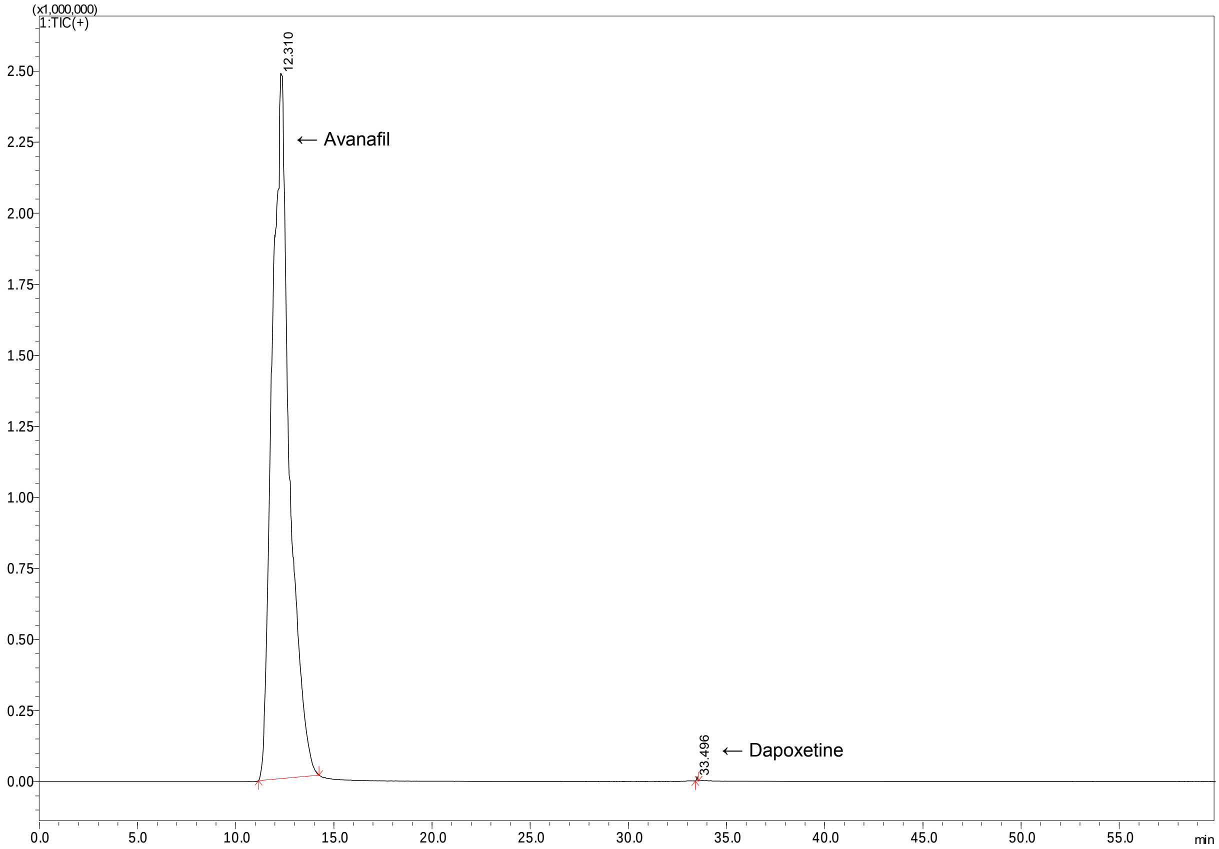

Figure S13. Assay TIC (+) chromatogram of TOP AVANA recorded using LC-MS/MS instrument (Analysis conditions are as follows: 0-15 min: 0.1% formic acid in water and 0.1% formic acid in acetonitrile (75:25, *v*/*v*, pH at 2.6); 15-60 min: 0.1% formic acid in water and 0.1% formic acid in acetonitrile (5:95, *v*/*v*, pH at 2.6). Retention times are 12.3 min for AVA and 33.49 min for dapoxetine).
